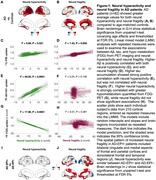# Neural circuit hyperexcitability, amyloid‐beta (Aβ), tau, and neurodegeneration in patients with early‐stage Alzheimer's disease

**DOI:** 10.1002/alz70855_097235

**Published:** 2025-12-23

**Authors:** Kamalini G Ranasinghe, Kiwamu Kudo, Faatimah Syed, Claire Yballa, Joel H Kramer, Bruce L. Miller, Katherine P Rankin, Paul Garcia, Heidi Kirsch, Keith Vossel, William J. Jagust, Gil D. Rabinovici, Srikantan S Nagarajan

**Affiliations:** ^1^ University of California San Francisco, San Francisco, CA, USA; ^2^ Memory and Aging Center, Weill Institute for Neurosciences, University of California, San Francisco, San Francisco, CA, USA; ^3^ Memory and Aging Center, UCSF Weill Institute for Neurosciences, University of California, San Francisco, San Francisco, CA, USA; ^4^ Department of Neurology, Memory and Aging Center, University of California San Francisco, San Francisco, CA, USA; ^5^ Memory and Aging Center, Weill Institute for Neurosciences, University of California San Francisco, San Francisco, CA, USA; ^6^ University of California Los Angeles, Los Angeles, CA, USA; ^7^ Neuroscience Department, University of California, Berkeley, Berkeley, CA, USA; ^8^ Memory and Aging Center, Weill Institute for Neurosciences, University of California, San Francisco (UCSF), San Francisco, CA, USA; ^9^ Department of Radiology and Biomedical Imaging, University of California, San Francisco, San Francisco, CA, USA

## Abstract

**Background:**

Neural circuit hyperexcitability is mechanistically linked to amyloid‐beta (Aβ) and tau in Alzheimer's disease (AD) mouse models. In AD patients, although epileptiform activity—the clinical phenotype of neural circuit hyperexcitability, occurs at a higher incidence, the relationship between Aβ, tau, and hyperexcitability, remains unclear.

**Method:**

Here, in a well characterized cohort of, biomarker confirmed, early‐stage AD patients (*n* = 82; including 45 CDR=0.5 and 37 CDR=1) we used magnetoencephalography (MEG) to quantify hyperexcitability and positron emission tomography (PET) to quantify Aβ, tau, and neurodegeneration. We estimated neural circuit hyperexcitability as related to two different levels of neuronal and synaptic function. First, excitatory‐inhibitory (E/I) imbalance within a given region may be the result of intrinsic processes including abnormal local synaptic activity. We termed this abnormal local synaptic integration ‘***Neural hyperactivity***’, and quantified it using spectral aperiodic slope (15‐50 Hz). Second, altered E/I may be the result of abnormal synaptic inputs from long‐range functional connections to a given region. We quantified this abnormal long‐rnage synaptic input integration using a previously well described metric—***Neural fragility*** (*Li et. al 2021 Nat Neurosceince*). All measures were estimated for 210 cortical regions (Brainnetome atlas).

**Result:**

We found that both neural hyperactivity and neural fragility were increased in AD patients compared to age‐matched controls (Fig‐1). Aβ accumulation correlated with both increased neural fragility and increased neural hyperactivity while tau accumulation distinctly correlated with increased neural hyperactivity but not with neural fragility. Neurodegeneration was tightly correlated to neural hyperactivity, but not to neural fragility. Importantly, we found that AD patients with epileptiform manifestations (**AD‐EPI+**), when compared to patients without epileptiform manifestations (**AD‐EPI−**), have significantly increased neural fragility despite similar levels of neural hyperactivity, suggesting that AD epileptic phenotype may represent additional vulnerabilities to Aβ related neural circuit hyperexcitability processes.

**Conclusion:**

Collectively our results demonstrate that neural circuit hyperexcitability in patients with AD is not a uniform expression and instead involves diverse manifestations of synaptic integration deficits and their distinct associations with tau and Aβ. Targeting differential manifestations hyperexcitability associated with Aβ and tau could guide interventions aimed at mitigating E/I imbalance to improve cognitive outcomes in patients with AD.